# 

**Published:** 1844-03

**Authors:** 


### CONTENTS

OF NO. III.

### ORIGINAL COMMUNICATIONS.

ESSAYS AND CASES.

Art. I.—Analetical Report of a Series of Experiments in Mesmeric Somniloquism, performed by an association of gentlemen. - - - - - - 189

Art. II.—Cases of Dyspepsia successfully treated on the purgative plan. By William M. Yandell, M.D., of Benton, Miss. - - - - - - - - 219

Art. III.—Extirpation of a large part of the Superior Maxillary Bone, on account of a Schirrous Tumor developed in the Antrum. By Dr. F. G. del Valle.—Translated from the Spanish by the Junior Editor. 223

REVIEWS.

Art. IV.—Lectures on the Principles and Practice of Physic; delivered at King's College, London. By Thomas Watson, M.D., Fellow of the Royal College of Physicians, Physician to the Middlesex Hospital, and formerly Fellow of St. John's College, Cambridge. 228

Art. V.—On the nature and treatment of Stomach and Renal Diseases; being an inquiry into the connexion of Diabetes, Calculus, and other affections of the Kidney and Bladder, with indigestion. By Wm. Prout, M.D., F.R.S., Fellow of the Royal College of Physicians. 250

SELECTIONS FROM AMERICAN AND FOREIGN JOURNALS.

Pathological Causes of Cyanosis. - - - - - - 267
Cure of Hydrocephalus by means of Hydriodate of Potassa. 269
Propriety of operating in cases of Cataract. - - 270
Tobacco in Hysteria and Spasmodic Stricture of the Uterus. 272
Diabetes Mellitus successfully treated by Ioduret of Iron. 273
Magneto-Electricity in various Diseases. - - - 274
Sulphur Waters on the Secretory Organs. - - - 276
Objections to the use of Pessaries. - - - - 277
Face-Ache. - - - - - - - - - - - - 277
The alleged infecundity of females born co-twins with males. 277
Sign of Pregnancy. - - - - - - - 278

ORIGINAL INTELLIGENCE.

Magneto-Electricity in Disease. - - - - - 279
Medical Institute of Louisville. - - - - - - 280
Summer Course of Medical Lectures. - - - - 281
Paris' Pharmacologia. - - - - - - - 281
Medical Department of the St. Louis University. - - 282
Magendie's Physiology, - - - - - - 283
Medical School Catalogues. - - - - - - - 283
Impeachment of Professor Hall, of Baltimore. - - 284
Books Received. - - - - - - - - - 284

